# Functional magnetic resonance imaging (fMRI) of attention processes in presumed obligate carriers of schizophrenia: preliminary findings

**DOI:** 10.1186/1744-859X-7-18

**Published:** 2008-10-03

**Authors:** Francesca Mapua Filbey, Tamara Russell, Robin G Morris, Robin M Murray, Colm McDonald

**Affiliations:** 1 The Mind Research Network, Albuquerque, New Mexico, USA; 2 Department of Psychology, Institute of Psychiatry, King's College, London, UK; 3 Department of Psychiatry, Institute of Psychiatry, King's College, London, UK; 4 Department of Psychiatry, National University of Ireland, Galway, Ireland

## Abstract

**Background:**

Presumed obligate carriers (POCs) are the first-degree relatives of people with schizophrenia who, although do not exhibit the disorder, are in direct lineage of it. Thus, this subpopulation of first-degree relatives could provide very important information with regard to the investigation of endophenotypes for schizophrenia that could clarify the often contradictory findings in schizophrenia high-risk populations. To date, despite the extant literature on schizophrenia endophenotypes, we are only aware of one other study that examined the neural mechanisms that underlie cognitive abnormalities in this group. The aim of this study was to investigate whether a more homogeneous group of relatives, such as POCs, have neural abnormalities that may be related to schizophrenia.

**Methods:**

We used functional magnetic resonance imaging (fMRI) to collect blood oxygenated level dependent (BOLD) response data in six POCs and eight unrelated healthy controls while performing under conditions of sustained, selective and divided attention.

**Results:**

The POCs indicated alterations in a widely distributed network of regions involved in attention processes, such as the prefrontal and temporal (including the parahippocampal gyrus) cortices, in addition to the anterior cingulate gyrus. More specifically, a general reduction in BOLD response was found in these areas compared to the healthy participants during attention processes.

**Conclusion:**

These preliminary findings of decreased activity in POCs indicate that this more homogeneous population of unaffected relatives share similar neural abnormalities with people with schizophrenia, suggesting that reduced BOLD activity in the attention network may be an intermediate marker for schizophrenia.

## Background

Imaging studies of attention processes in people with schizophrenia have reported widespread functional abnormalities in the attention network (Andreasen, 1995 [1]; Goldman-Rakic, 1988 [2]). For example, using an oddball paradigm to assess sustained attention, a functional magnetic resonance imaging (fMRI) study found decreased activity in superior temporal and frontal gyri in addition to the cingulate cortex and thalamus [3]. Similarly vast areas of decreased activity during a target detection task were found in patients with schizophrenia that included areas in the limbic cortex, striatum, anterior cingulate gyrus and prefrontal cortex [4]. During a selective attention task, decreased activation was also found in several areas of the dorsolateral prefrontal cortex (DLPFC), anterior cingulate in addition to parietal areas [5]. Evoked related potential recordings have also shown reduced hippocampal activation, in addition to reductions in the anterior cingulate gyrus and basal ganglia during orienting of attention [6].

Because attention deficits are a key and a stable feature of schizophrenia [7], those who share the same genes for schizophrenia may also show similar patterns. However, findings from neuropsychological studies of those who are at genetic risk for schizophrenia have been inconsistent [8,9], partly because these studies often rely on the assumption of elevated genetic risk for schizophrenia based on population levels of chance. For example, a study of 20 subjects with 1 relative with schizophrenia will effectively deal with a risk increase from 1% to 2–4%, which does not necessarily reflecting strong genetic load. Another possibility for these inconsistencies may be due to the subtlety of the deficits that make them undetectable behaviourally. Neurophysiological measures of attention, such as eye tracking, may be a better indicator of attention deficits as they are more proximal to the biological mechanisms. For example, eye tracking studies have suggested that abnormalities in antisaccades and smooth pursuit are possible endophenotypes for schizophrenia [10,11]. In addition, electrophysiological abnormalities (i.e., late P300) during target detection despite normal task performance has been reported in first-degree relatives of people with schizophrenia [12,13]. This is evidence to suggest that these enduring traits related to attentional processing are also present in those who are genetically related to people with schizophrenia.

Neuroimaging studies of high-risk populations have recently begun to investigate a subgroup of relatives of patients with schizophrenia, sometimes referred to as presumed obligate carriers (POCs) in order to segregate a more homogeneous group [14,15]. These individuals are the first-degree relatives of people with schizophrenia who, although they do not manifest the disorder, are in direct lineage of it. Thus, POCs are presumed to transmit the genes for schizophrenia. Structural neuroimaging findings have suggested volumetric differences between POCs and healthy controls [14,16]. For example, it was found that in contrast to healthy, non-carrier siblings, POCs have reduced amygdalohippocampal complex volumes [14]. At present, we are aware of only one published functional neuroimaging study of POCs. Using positron emission tomography (PET), Spence *et al*. tested neural function associated with verbal fluency. The authors reported that the POCs had a pattern of frontal activity similar to that of the individuals with schizophrenia (i.e., reduced bilateral activation of the dorsolateral prefrontal cortex), and that it is qualitatively different from that of controls. The authors suggested that this abnormality may be due to either a loss of asymmetry or 'hyperinnervation' between the prefrontal cortices [17].

The present study attempts to extend the literature on POCs of schizophrenia by examining the neural correlates of attentional abnormalities using fMRI. fMRI provides the ability to investigate the underlying processes that may be dysfunctional despite intact cognitive behaviour. The aims of this study were: (1) to contribute to the growing literature on neural abnormalities present in people who have unexpressed genetic predisposition to schizophrenia, and (2) to demonstrate the utility of fMRI in characterising intermediate phenotypes for schizophrenia.

Because attention deficits have been widely reported as possible intermediate phenotypes between genotype and clinical symptoms of schizophrenia (i.e., present in those who are at high genetic risk), we chose to investigate the neural mechanisms that are associated with attention processes in the POCs of schizophrenia. Using fMRI, we measured sustained, selective and divided attention processes in order to investigate whether the genetic predisposition to schizophrenia is associated with differential neural activity that could be used as biomarker for schizophrenia. Based on previous studies, we expected that abnormalities in a widely distributed network of brain regions including the parietal, prefrontal and temporal cortices, the hippocampus, the reticular formation, and the striatum that underlie attention would show a differential response in POCs [18]. Prefrontal areas, particularly the DLPFC and ventrolateral prefrontal cortex are important areas for attention processes (i.e., sustained, selective, divided), in addition to executive functioning processes (i.e., inhibition and working memory) [19-22]. In addition to the prefrontal cortex, the anterior cingulate gyrus has also been suggested to be involved in target detection, response selection and inhibition, error detection, performance monitoring, and motivation [23-25]. The reticular formation, which sends input to the thalamus, appears to modulate attention and filter interfering stimuli [26], while the hippocampus has been suggested to play a role in attention, possibly as a 'comparator' by processing varieties of input and resolving conflict [18].

We included POCs in this study who are healthy relatives who lie between two affected generations (e.g., have both a parent and a child with schizophrenia), thus increasing the likelihood that these individuals carry genes linked to schizophrenia. Because the existing literature on POCs report that this population shares similar neural abnormalities present in those with schizophrenia, we expected to find abnormalities in neural activity associated with attention processes in our group of POCs [17,27,28].

## Methods

### Participants

Eight POCs and eight healthy participants gave informed consents and completed the fMRI study. These participants comprise a subset of unaffected relatives of patients with schizophrenia. The recruitment and clinical assessments of whom have been described in detail previously [29]. Participants were selected based on ability to perform the attention conditions offline and a negative screening for MRI contraindications. All participants were right-handed Caucasians. Due to the small sample size non-parametric analyses explored differences in demographic variables between the groups. Participants performed a practice test outside of the scanner and proceeded to the scanning session after demonstrating their understanding of, and ability to perform, the task. Two of the POCs could not complete the scanning protocol (due to discomfort) and had to be excluded from further analyses. Out of those who completed the scanning protocol successfully, we found no significant difference using the Mann-Whitney U test (for age and IQ) and Chi-square test (for gender) analyses in age, gender or IQ as measured by the revised Wechsler Adult Intelligence Scale (WAIS-R) between POCs and healthy participants (p > 0.05; see Table 1).

**Table 1:** Summary of demographic variables for participants

	**POC**	**Healthy participants**
n	6	8
Mean age (range)	53 (49–59)	41 (18–60)
N (males/females)	2/4	5/3
Mean IQ* (SD)	111 (16)	115 (11)

The two groups did not differ in any of these measures (p > 0.05). POC, presumed obligate carriers.

*As measured by the revised Wechsler Adult Intelligence Scale (WAIS-R), short version.

### Assessments

**Figure 1:**
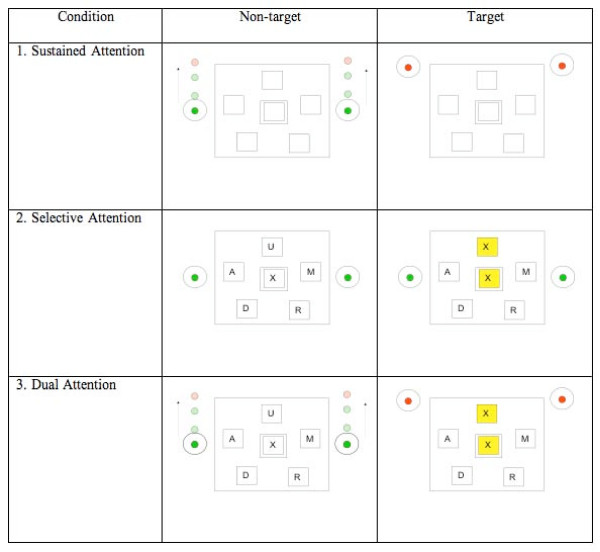
**Schematic of task visual presentations per task**. An example of the screen as viewed by the participants during the active or 'on' period of (1) sustained attention task, (2) selective attention task and (3) dual attention task. During the sustained attention task, the circles move vertically and the colour in the centre of the circles change. The participants are asked to respond when the colour changes from green (non-target) to red (target). During the selective attention task, participants are asked to respond when any of the five surrounding letters match the one in the middle (target). Positive feedback is given in the form of highlighted matches (target). During the dual attention task, participants are asked to simultaneously monitor the circles and respond to letter matches at the same time as previously described.

Participants performed a modified version of a divided attention paradigm previously described by Necka [30]. Reaction times were recorded as a measure of task performance during the following tasks presented in separate runs (see Figure 1):

1. Sustained attention task: participants were asked to monitor the synchronous vertical movements of two circles (one on each side of the screen) by pressing a button on a response pad. The objective was to respond to colour cues located in the centre of the circles (change from green to red) by either pressing the button (when circles are on top of screen) to bring circles down or releasing the depressed button (when circles are on bottom of the screen). The top and bottom thresholds were equally set at 1/3 of the test screen (i.e., circles turn red at the top 1/3 of the screen and at the bottom 1/3 of the screen). The speed at which the circles moved was unpredictable. Reaction times to the colour cues were recorded.

2. Selective attention task: participants were presented with letters to which they had to respond to by button pressing if the target letter (located in the centre of the screen) matched any of the five flanking letters (see Figure 1). The target letter (middle square) changed every 20 s and the five letters surrounding target letter changed every 2 s. The target rate was 50%. Reaction times to correct matches were recorded.

3. Dual attention task: participants were asked to simultaneously perform the sustained and selective attention tasks as described above.

The tasks were presented in the same order across all subjects as follows: sustained attention task, selective attention task, and dual attention task. Tasks were presented using a block design with 30-s 'on' trials being the active periods of the tasks and the 30-s 'off' trials with participants instructed to 'rest' while maintaining visual attention to the screen. The 'off' trials for all conditions had the same visual stimuli as the 'on' trials for each task (see Figure 1). Only the participants who were able to perform the tasks outside of the scanner were included in the study.

### Data acquisition

fMRI data were acquired at the Maudsley Hospital, King's College, London, UK. Gradient-echo, echo-planar MR images were acquired using a 1.5 Tesla General Electric Signa System (General Electric, Milwaukee, WI, USA). A total of 100 T2-weighted MR images depicting blood oxygenated level dependent (BOLD) contrast were acquired at each of 14 non-contiguous near axial planes (7 mm thick with 0.7 mm slice skip; in-plane resolution = 3 mm) parallel to the anterior commissure-posterior commissure line at an echo time of 40 ms and a repetition time of 3000 ms. Head movement was minimised by foam padding with the head coil and a restraining band placed across the forehead. In the same session, a 43-slice high-resolution inversion recovery, gradient-echo, echo-planar image series of the whole brain was again acquired parallel to the intercommissural plane. This latter data set allows improved visualisation of the anatomy while maintaining any geometric distortion inherent within the echo-planar imaging (EPI) methodology. Because slight subject motion during image acquisition can cause changes in T2-weighted signal density unrelated to changes in oxyhaemoglobin/deoxyhaemoglobin ratio, a procedure adopted from Friston *et al*. was used to correct the effects of motion prior to any further analysis of the images [31].

### Data analysis

Using SPSS v.11 (SPSS, Chicago, IL, USA), response times were log transformed in order to normalise the distribution and analysed using Mann-Whitney U tests to compare differences between the groups.

Imaging data analyses were carried out using FEAT (fMRI Expert Analysis Tool) Version 5.43, part of FSL (FMRIB's Software Library; ) using the following pre-statistics processing: non-brain removal using brain extraction tool (BET; Smith, 2002); spatial smoothing using a Gaussian kernel of full width at half maximum (FWHM) 8 mm; mean-based intensity normalisation of all volumes by the same factor; high-pass filter cut-off of 100 s; high-pass temporal filtering (Gaussian-weighted low spatial frequency straight line fitting, with sigma = 50.0s). Time series statistical analysis was carried out using FILM (FMRIB's Improved Linear Model) with local autocorrelation correction (Woolrich, 2001). Regressors were created by convolving the stimulus timing files with a double gamma variate haemodynamic response function. A multiple linear regression analysis was performed to estimate the haemodynamic parameters for different explanatory variables (i.e., active and rest periods) and a corresponding t statistic indicates the significance of the activation of the stimulus. Contrast maps were created by contrasting the active and rest periods for all three conditions. These maps were then registered to a high-resolution image using fMRI linear image registration tool (FLIRT; Jenkinson 2001, 2002). Group analyses were carried out using FLAME (FMRIB's local analysis of mixed effects) (Beckmann 2003 [32], Woolrich 2004 [33]).

The resulting activation differences were then cluster thresholded to correct for multiple comparisons at z = 2.8 (p < 0.05). Cluster inference in FSL uses a familywise error (FWE) correction, thus, p = 0.05 controls the chance of one or more false positive clusters anywhere in the brain. Finally, MRI3dx  was used to determine the corresponding labels for the areas of significant difference.

## Results

Table 2 summarises the mean reaction times for each condition per group. The POCs were significantly slower in their reaction times than the normal controls (p = 0.03) during the letters task. The groups did not differ in the balls or dual task.

**Table 2:** Behaviour response data

	**POC mean (SD)**	**Healthy participants mean (SD)**
Circles:		
Sustained attention	0.46 (0.47)	0.31 (0.007)
Dual attention	0.73 (0.27)	0.61 (0.34)
Letters:		
Selective attention	1.18 (0.17)	1.08 (0.19)
Dual attention	1.31 (0.23)*	2.43 (0.75)*

This table outlines the mean reaction times (in seconds) of the presumed obligate carriers and the healthy participants to the circles during the sustained attention task and the dual attention task, and the letters during the selective attention task and the dual attention task. POC, presumed obligate carriers.

* p < 0.05

### Sustained attention task

**Figure 2:**
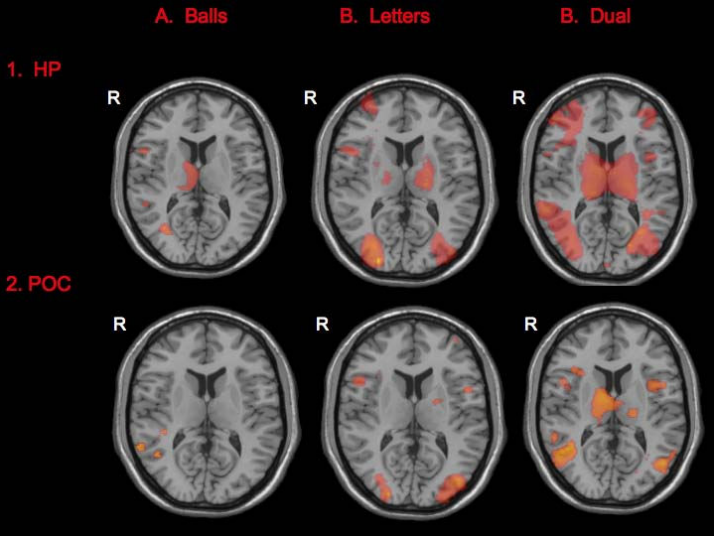
**Activation maps per group per attention task**. The attention tasks elicited greater response in widespread areas in both the healthy participants (HP) and the presumed obligate carriers (POCs). There were no areas with greater activation during rest compared to the attention trials in either group. Right hemispheric activations are on the left side of the images. For ease of comparison across groups and across tasks, activations are displayed on axial images and thresholded at z = 3.0.

In the healthy participants group, sustained attention processes were associated with a widespread pattern of increased activity in bilateral inferior and superior parietal lobe, bilateral middle occipital gyri, right (R) inferior occipital gyrus, bilateral pre- and post-central gyri, bilateral superior, middle, inferior frontal gyri, R medial frontal gyrus, R middle temporal gyri, left (L) superior and inferior temporal gyri, L culmen, left precuneus, R thalamus, R subcallosal gyrus, L cingulate gyrus, L caudate and L precuneus (cluster corrected p < 0.05, z = 2.8) (Figure 2). There were no areas where rest was greater than the periods of sustained attention.

In the POCs, significantly increased activity was found in the following regions during sustained attention trials compared to rest (listed in descending order of size): bilateral middle temporal gyri, bilateral superior and inferior frontal gyri, L middle frontal gyrus, R medial frontal gyrus, bilateral post-central gyri, R precentral gyrus, L insula, R cingulate gyrus, bilateral thalamus, R fusiform gyrus, bilateral culmen, bilateral inferior and superior parietal lobe, bilateral inferior occipital gyri, L superior and inferior temporal gyri, and R middle temporal gyrus during the sustained condition (cluster corrected p < 0.05, z = 2.8) (Figure 2). There were no areas where rest was greater than the periods of sustained attention.

**Figure 3:**
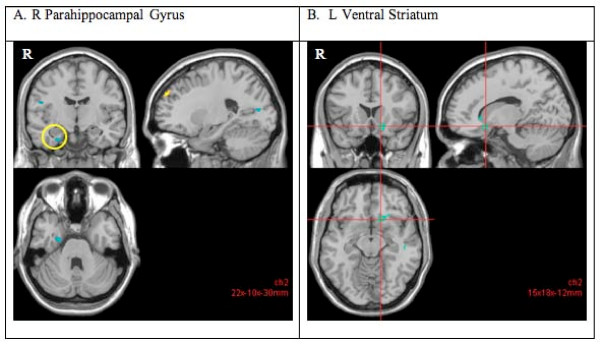
**Decreased blood oxygenated level dependent (BOLD) response (cluster corrected p < 0.05, z = 2.8) in the presumed obligate carriers (POCs) compared to the healthy participants during the sustained attention (vs rest) task**. The POCs indicated decreased activity in the parahippocampal gyrus (A) and ventral striatum (B) compared to the healthy participants. Right hemispheric activations are on the left side of the images.

When the two groups were compared, the POCs had significantly less activation compared to the healthy participants during sustained attention in several areas in the fronto-temporo-parietal areas, that included the cingulate and parahippocampal gyri (see detailed list in Table 3 and illustrated in Figure 3) (cluster corrected p < 0.05, z = 2.8).

**Table 3:** Differences in blood oxygenated level dependent (BOLD) response between the two groups during the sustained attention, selective attention and divided attention tasks

	**Max z-score**	**TLRC co-ordinates**	**BA**
Sustained attention:			
HP > POC			
R inferior parietal lobe	3.8	[42 -58 48]	7
R subcallosal gyrus	3.7	[16 20 -13]	11
R superior parietal lobe	3.6	[30 -64 54]	7
R mid-occipital gyrus	3.3	[38 -92 -4]	18
R mid-occipital gyrus	3.2	[28 -96 1]	18
R mid-temporal gyrus	3.2	[48 -20 -12]	21
R posterior cingulate gyrus	3.1	[26 -64 12]	31
R superior frontal gyrus	3.0	[18 62 1]	10
R lingual gyrus	3.0	[32 -60 -5]	19
R parahippocampal gyrus/entorhinal cortex	2.9	[24 -10 -30]	28
L ventral striatum	2.9	[-17 11 -9]	-
L cuneus	2.9	[-24 -78 12]	18
R precentral gyrus	2.4	[50 -8 22]	43
R precentral gyrus	2.8	[34 22 38]	9
R lingual gyrus	2.8	[4 -94 -5]	18
POC > HP			
L superior temporal gyrus	3.5	[-64 -52 8]	21
L superior frontal gyrus	2.9	[-22 56 31]	9
R mid-temporal gyrus	2.8	[54 -74 18]	19
Selective attention:			
HP > POC			
R cingulate gyrus	4.2	[24 -46 38]	31
R cuneus	4.1	[26 -88 8]	18
R parahippocampal gyrus	3.7	[38 -48 -9]	19
R precuneus	3.3	[14 -70 36]	7
R mid-frontal gyrus	3.3	[36 56 1]	10
R insula	3.0	[40 -4 -5]	13
R fusiform gyrus	3.0	[34 -72 -20]	19
R caudate	3.0	[14 6 20]	-
L insula	3.0	[-44 -10 0]	13
R globus pallidus	2.9	[22 -14 -2]	-
R inferior frontal gyrus	2.9	[40 18 -12]	47
POC > HP			
R mid-temporal gyrus	4.3	[72 -28 -4]	21
L mid-temporal gyrus	4.2	[-46 -72 20]	39
R mid-temporal gyrus	3.7	[70 -42 0]	21
L mid-frontal gyrus	3.5	[-18 66 26]	10
L superior frontal gyrus	3.4	[-24 42 34]	10
L supramarginal gyrus	3.0	[-56 -42 36]	40
R parahippocampal gyrus	3.0	[30 -24 -18]	36
R mid-temporal gyrus	3.0	[56 0 -34]	21
L mid-frontal gyrus	3.0	[-54 28 30]	9
Dual attention:			
HP > POC			
L inferior frontal gyrus	4.0	[-24 14 -22]	47
L mid-temporal gyrus	3.8	[-40 -44 1]	19
L inferior parietal lobe	3.8	[-32 -40 26]	13
L medial frontal gyrus	3.6	[-6 40 44]	8
L superior temporal gyrus	3.6	[-46 20 -28]	38
R medial frontal gyrus	3.7	[10 32 46]	8
R inferior frontal gyrus	3.3	[54 42 12]	46
R mid-occipital gyrus	3.3	[30 -72 4]	18
L parahippocampal gyrus	3.2	[-20 -10 -23]	35
R hypothalamus	3.1	[8 -6 -9]	-
R precuneus	3.1	[24 -50 44]	7
L cingulate gyrus	3.0	[-22 -22 40]	24
L parahippocampal gyrus	2.8	[-22 -34 -9]	30
R mid-frontal gyrus	2.8	[42 62 -4]	10
POC > HP			
R mid-temporal gyrus	3.3	[58 -68 18]	39
L superior temporal gyrus	3.1	[-40 -6 -12]	21
L culmen	3.0	[-20 -40 -22]	-

Foci of significantly greater activation during the attention tasks when the groups were compared (only peaks with z > 2.8 reported here). Localisation, Tailarach co-ordinates (TLRC) and corresponding Brodmann areas (BA) represents the voxel with the maximum z-score; HP, healthy participants; L, left; POCs, presumed obligate carriers; R, right.

### Selective attention task

During the selective attention trials (vs rest), the healthy participants had increased activity in the following areas (listed in descending order of size): L superior temporal gyrus, bilateral cingulate gyri, right fusiform gyrus, R middle occipital gyrus, bilateral inferior, frontal gyri, L superior and inferior frontal gyri, bilateral precuneus, L superior and inferior parietal lobe, R culmen, L pre-central gyrus, R rectal gyrus, (cluster corrected p < 0.05, z = 2.8) (Figure 2). There were no areas where rest was greater than the periods of selective attention.

In the POCs, there was increased activity in the following regions during the selective attention trials (listed in descending order of size): bilateral precuneus, bilateral middle occipital gyri, R fusiform gyrus, bilateral inferior and middle frontal gyri, R medial frontal gyrus, bilateral inferior and middle temporal gyrus, L supramarginal gyrus, R culmen, L cingulate gyrus, R culmen, R inferior and superior parietal lobe, R rectal gyrus, and R thalamus during the selective attention trials compared to the rest trials (cluster corrected p < 0.05, z = 2.8) (Figure 2). There were no areas where rest was greater than the periods of selective attention.

**Figure 4:**
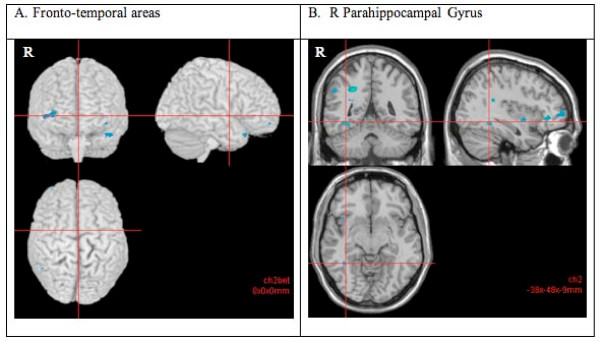
**Decreased blood oxygenated level dependent (BOLD) response (cluster corrected p < 0.05, z = 2.8) in the presumed obligate carriers (POCs) compared to the healthy participants during the selective attention (vs rest) task**. The POCs indicated decreased activity in the frontal (right mid- and inferior frontal gyri, right cingulate gyrus) and temporal cortices (A), and R parahippocampal gyrus (B) compared to the healthy participants. Right hemispheric activations are on the left side of the images.

When the two groups were compared, the POCs had decreased activity in the basal ganglia, in addition to fronto-temporal areas during selective attention trials (cluster corrected p < 0.05, z = 2.8) (see detailed list in Table 3 and Figure 4).

### Dual attention task

In the healthy participants, dual attention (vs rest) was associated with increased BOLD response in the following areas (listed in descending order of size): bilateral cingulate gyri, L culmen, bilateral inferior and middle frontal gyri, R inferior parietal lobe, L lentiform nucleus, R medial frontal gyrus, L paracentral lobule, R parahippocampal gyrus, bilateral precentral gyrus, bilateral precuneus, L superior frontal gyrus, L middle occipital gyrus, bilateral middle and superior temporal gyrus, L supramarginal gyrus and bilateral thalamus in the healthy participants (cluster corrected p < 0.05, z = 2.8) (Figure 2). There were no areas where rest was greater than the periods of dual attention.

In the POCs, dual attention (vs rest) was associated with increased BOLD activity in the following (listed in descending order of size): bilateral cingulate gyri, R inferior occipital gyrus, bilateral culmen, bilateral superior middle and temporal gyri, bilateral middle occipital gyrus, bilateral thalamus, bilateral precuneus, L middle and inferior frontal gyri, R medial frontal gyrus, L lentiform nucleus, bilateral inferior parietal lobe, R parahippocampal gyrus, and L pre- and post-central gyrus (cluster corrected p < 0.05, z = 2.8) (Figure 2). There were no areas where rest was greater than the periods of dual attention.

**Figure 5:**
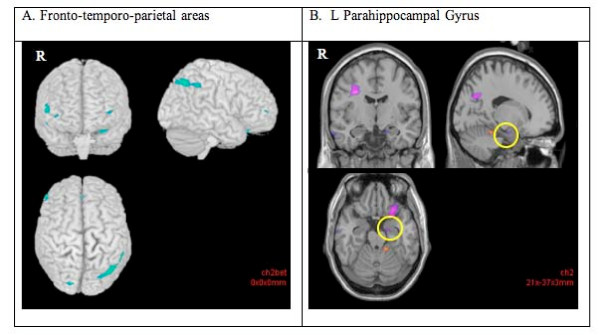
**Decreased blood oxygenated level dependent (BOLD) response (cluster corrected p < 0.05, z = 2.8) in the presumed obligate carriers (POCs) compared to the healthy participants during the dual attention (vs rest) task**. The POCs indicated decreased activity compared to the healthy participants in the frontal (right and left inferior and medial frontal gyri, and right middle frontal gyrus), temporal (left mid-temporal gyrus) and parietal cortex (right precuneus, left inferior parietal lobule) (A), and in the left parahippocamal gyrus (B). Right hemispheric activations are on the left side of the images.

When the groups were compared, several areas of significantly decreased activity were found in the POCs compared to the healthy participants (cluster corrected p < 0.05, z = 2.8). These included frontal, cingulate, temporal and occipital areas, and, the hypothalamus (see Table 3 and Figure 5).

## Discussion

This study reports preliminary findings from a cohort of individuals who are the presumed obligate carriers of genes linked to schizophrenia. The performance of this group was compared to that of healthy age/gender/IQ matched individuals on three measures of attention: sustained, divided and dual attention. We hypothesised that if these individuals are carriers of genes that code for schizophrenia then their performance on these tasks would differ from healthy participants and be more similar to the reported findings in those with schizophrenia. As predicted, the POCs showed altered neural activation patterns relative to the healthy participants during sustained, selective and divided attention as measured by the BOLD response. Compared to the healthy participants, the POCs demonstrated a general decreased pattern of activation during the three attention tasks despite unimpaired task performance.

Our findings showed that POCs have altered neural function in the attention network that is similar to the abnormalities previously reported in patients with schizophrenia. While differences in activation appeared to be widespread in all three tasks of attention, the POCs had attenuated response in prefrontal areas and parahippocampal gyrus/entorhinal cortex during all three tasks. Prefrontal abnormalities, including those in the anterior cingulate gyrus, have been suggested to be the best indicator of genetic vulnerability to schizophrenia as they have been found in the unaffected relatives of those with schizophrenia [34,35]. Temporal lobe abnormalities, particularly in the medial portion, have also often been reported in people with schizophrenia and have been associated with the psychotic and neuropsychological symptoms of schizophrenia [36]. Both structural [37] and functional [34,38] temporal lobe abnormalities have also been reported in the relatives, therefore, indicating that these may be an indicator of risk for schizophrenia. In the present study, decreased activity in the parahippocampal gyrus of the POCs was observed during all three conditions of attention. The parahippocampal gyrus surrounds the hippocampus and lies in the medial temporal lobes of the brain. It has been argued that a disordered 'hippocampal formation' (i.e., hippocampus and parahippocampal gyrus) is the fundamental abnormality in the pathophysiology of schizophrenia based on decreased size, reduced *N*-acetyl aspartate, altered metabolic/synaptic activity, presence of memory deficits (attributable to hippocampal dysfunction), and alterations in receptors [39-42]. In addition to the role of the hippocampus in memory formation, it has also been suggested that it plays a crucial role in integrating multimodal sensory inputs, and in resolving conflicts between expectancies and the current perception [43,44]. This is in accord with the role of the hippocampus in encoding attention proposed by Mirsky *et al. *[18].

The widespread nature of disrupted neural activity in the present group of POCs provides further support for the idea that schizophrenia is an inter-region disconnectivity syndrome [45]. Since frontal abnormalities are the cardinal feature in the pathophysiology of schizophrenia, it is not surprising that parietal and temporal abnormalities exist because reciprocal connections exist between the PFC, parietal, temporal and cingulate cortices [46]. In a recent study that investigated functional connectivity in schizophrenia, it was found that even during rest (i.e., without a directed task), disconnectivity was found globally in those with schizophrenia [45]. Of the 177 connectivities investigated, 158 showed decreased activity in the individuals with schizophrenia compared to the healthy participants. Moreover, functional connectivity abnormalities have also been found in individuals with high genetic risk for schizophrenia, such that altered connectivity was found between the parietal and prefrontal network, and the prefrontal cortex and cerebellum [47].

Our findings also showed that during sustained and selective attention, the POCs had less right hemispheric activation in the fronto-temporo-parietal network. Posner and Petersen posited the notion of a right-lateralised vigilance system that is due to greater innervation of ascending noradrenergic pathways in the right hemisphere [48]. In this context, decreased activity in the POCs may reflect hypofunction of noradrenergic pathways leading to hypovigilance and distractibility. Dysfunctional noradrenergic pathways, in addition to abnormalities in dopaminergic pathways, have also been proposed in schizophrenia [49] and have been targets for more novel pharmacotherapies (e.g., quetiapine) [50]. Reduced BOLD signal in these regions is in accord with the frequently reported hypofrontality in schizophrenia. Despite inconsistencies in the literature, studies have generally provided evidence for attenuation of neural activity in the frontal cortex of people with schizophrenia [51]. Thus, our findings of significantly decreased activity in POCs in several areas of the prefrontal cortex during all three conditions are consistent with previous findings [52]. Of note, although disturbances in the frontal lobes have been the hallmark of schizophrenia, hypofunction has also been reported in the parietal and temporal lobes [53].

While we observed a general decrease in regions that underlie attention in the POCs during attention processes, these findings must be interpreted in light of some areas that show increased response (i.e., temporal regions). Enhanced activity in conjunction with hypofunction in the attention network despite unimpaired behaviour performance may indicate recruitment strategies in the POCs. Recruitment or compensatory mechanisms in schizophrenia have been previously reported in the literature [13,54]. For example, it was found that during a working memory task, depending on task demands, greater activation in the PFC was found along with decreased activation in the posterior parietal cortex and vice versa [55]. It was suggested that function in the PFC was dependent on availability of other areas within the network. Additionally, the idea that neuroplastic processes may also be responsible cannot be discounted. It has been proposed that structural abnormalities may trigger regeneration or re-organisation of function [56]. Network modelling or similar techniques were beyond the scope of this report, but are important for future studies.

Lastly, although our findings confirm our hypothesis and are in accord with the current literature on POCs of schizophrenia, interpretation must be made with caution given our small sample size. Although the implications of having a small sample size pertains to generalisability of these findings, our findings are in concordance with previous neuroimaging studies in POCs with very small sample sizes, e.g. 6 for Steel *et al. *(2002) [14]; 11 for Chua *et al. *(2000) [57]; 9 for Sharma *et al. *(1999) [58]; 10 for Spence *et al. *(2000) [17]. Nevertheless, because this is the first fMRI study of POCs and the first to use this task, replication of these findings in a larger cohort of POCs is necessary in order to validate these results.

## Conclusion

These preliminary findings suggest the presence of abnormal patterns of neural activity in the POCs despite unimpaired performance during tasks of attention. Altered BOLD response was observed in fronto-temporo-parietal regions that have been shown to subserve attention processes. Our findings in the POCs during attention processes parallel those previously reported in people with schizophrenia, and may reflect compensatory mechanisms necessary to successfully attend to stimuli. In addition to replication of these findings, future research should also examine whether this abnormality is the cause, consequence, or compensation for the pathophysiology of schizophrenia [59] and how this may translate to functional disconnectivity.

## Competing interests

The authors declare that they have no competing interests.

## Authors' contributions

FF conceived of the study, collected, analysed and interpreted the data, and drafted the manuscript. RGM made contributions to the design of the study. TR provided assistance in the image analyses. CMcD provided the clinical diagnoses, screening and categorisation of the participants. RMM made substantial contributions in the drafting and revision of the manuscript. All authors read and approved the final manuscript.
